# COVID-19 vaccine effectiveness against in-hospital mortality in immunocompromised older adults: a Brazilian hospital surveillance study, 2024

**DOI:** 10.1590/1980-549720260041

**Published:** 2026-08-17

**Authors:** Rodrigo Poubel Vieira de Rezende, João Felipe Nicolaci Pimentel, Bruno Santos Caxias, Caio Moreira Salgueiro, Lucas Tanikawa de Oliveira, Luís Fernando de Araújo Santos, Caroline Pimentel Pessanha, Vitoria Xavier Tracierra, Michael Gunter Simão, Mario Gustavo de Aranda Pacheco, Vitor Alves Cruz, Luiz Eduardo da Costa Oliveira

**Affiliations:** I Universidade Federal Fluminense, Faculty of Medicine – Niterói (RJ), Brazil.; II Universidade Federal Fluminense, Institute of Computing – Niterói (RJ), Brazil.; III Universidade Federal de Goiás, Faculty of Medicine – Goiânia (GO), Brazil.

**Keywords:** Immunocompromised patients, Elderly, COVID-19, COVID-19 vaccines, Mortality, Pacientes imunocomprometidos, Idoso, COVID-19, Vacinas contra a COVID-19, Mortalidade

## Abstract

**Objective:**

The study evaluated the association between COVID-19 vaccination and in-hospital mortality among immunocompromised older adults hospitalized for COVID-19 in Brazil during the 2024 epidemiologic year.

**Methods:**

This retrospective hospital-based surveillance study used data from the Brazilian SIVEP-Gripe database. All hospitalized cases of COVID-19-related severe acute respiratory syndrome in immunocompromised individuals aged ≥60 years reported during epidemiological weeks 1–52 of 2024 were included. Multivariable logistic regression was used to estimate odds ratio (OR) and 95% confidence interval (95%CI) for the association between COVID-19 vaccination (number of doses) and in-hospital mortality, adjusting for clinical and sociodemographic covariates.

**Results:**

Among 474 patients, in-hospital mortality was 36% (171/474). Only 56 (12%) were unvaccinated. Compared with unvaccinated individuals, adjusted ORs for mortality were 0.67 (95%CI 0.32–1.39) for 1–2 doses, 0.67 (95%CI 0.31–1.42) for 3 doses, 0.54 (95%CI 0.26–1.15) for 4 doses, and 0.56 (95%CI 0.26–1.17) for 5–6 doses. Mechanical ventilation (OR 12.55; 95%CI 6.39–26.33) and lower educational attainment (elementary education: OR 2.94; 95%CI 1.39–6.44) were independent predictors of death. COVID-19 vaccination was not significantly associated with reduced in-hospital mortality among immunocompromised older adults hospitalized for the disease in Brazil in 2024. However, all adjusted ORs were below unity, suggesting a trend toward protection with increasing doses.

**Conclusions:**

The strong association of mortality with mechanical ventilation and social determinants highlights the need for timely boosters and comprehensive preventive strategies for this vulnerable population.

## RESUMO

**Objetivo:**

O estudo avaliou a associação entre vacinação contra COVID-19 e mortalidade hospitalar entre idosos imunocomprometidos hospitalizados por COVID-19 no Brasil durante o ano epidemiológico de 2024.

**Métodos:**

Trata-se de um estudo retrospectivo de vigilância hospitalar que utilizou dados do Sistema de Informação da Vigilância Epidemiológica da Gripe (SIVEP-Gripe). Foram incluídos todos os casos hospitalizados de síndrome respiratória aguda grave relacionada à COVID-19 em indivíduos imunocomprometidos com idade ≥60 anos notificados entre as semanas epidemiológicas 1–52 de 2024. Utilizou-se regressão logística multivariada para estimar razão de chance (OR) e intervalo de confiança de 95% (IC95%) para a associação entre vacinação contra COVID-19 (número de doses) e mortalidade hospitalar, ajustando-se para covariáveis clínicas e sociodemográficas.

**Resultados:**

Entre os 474 pacientes incluídos, a mortalidade hospitalar foi de 36% (171/474). Apenas 56 (12%) não eram vacinados. Em comparação aos não vacinados, os ORs ajustados para mortalidade foram de 0,67 (IC95% 0,32–1,39) para 1–2 doses, 0,67 (IC95% 0,31–1,42) para 3 doses, 0,54 (IC95%: 0,26–1,15) para 4 doses e 0,56 (IC95% 0,26–1,17) para 5–6 doses. Ventilação mecânica (OR 12,55; IC95% 6,39–26,33) e menor escolaridade (ensino fundamental: OR 2,94; IC95% 1,39–6,44) foram preditores independentes de óbito. A vacinação contra COVID-19 não se associou significativamente à redução da mortalidade hospitalar entre idosos imunocomprometidos hospitalizados pela doença no Brasil em 2024. Entretanto, todos os OR ajustados foram inferiores a 1, sugerindo tendência de proteção com o aumento do número de doses.

**Conclusões:**

A forte associação da mortalidade com ventilação mecânica e determinantes sociais reforça a necessidade de reforços vacinais oportunos e estratégias preventivas abrangentes para essa população vulnerável.

## INTRODUCTION

Immunocompromised individuals are at increased risk of severe outcomes from Coronavirus Disease 2019 (COVID-19), including death^1^. In response to this heightened vulnerability, many countries prioritized this population for COVID-19 vaccination, frequently adopting immunization strategies distinct from those recommended for the general population^2-4^.

Although available evidence suggests that COVID-19 vaccines confer protection against severe outcomes, including death, among immunocompromised individuals^5^, their effectiveness specifically in reducing COVID-19-related in-hospital mortality remains uncertain^6^. To date, only one population-based study has evaluated mortality among immunocompromised individuals hospitalized for COVID-19, and it found no significant difference in the odds of death between vaccinated and unvaccinated patients^6^.

Given the limited real-world evidence on the impact of COVID-19 vaccination on mortality among immunocompromised individuals hospitalized with the disease, this study aimed to examine the association between the vaccination and in-hospital mortality in immunocompromised older adults — a population at high risk for severe outcomes and a public health priority for vaccination.

## METHODS

This was an observational, retrospective, hospital-based surveillance study using data from the Brazilian Influenza Epidemiological Surveillance Information System (SIVEP-Gripe), a national registry maintained by the Brazilian Ministry of Health to document hospitalized cases of severe acute respiratory syndrome (SARS) in Brazil, including COVID-19. The analysis comprised cases reported during epidemiological weeks 1–52 of 2024 and used a publicly available, de-identified dataset obtained from OpenDATASUS (https://opendatasus.saude.gov.br) and accessed on March 29, 2025.

All records of COVID-19-related SARS resulting in hospitalization during epidemiological weeks 1–52 of 2024 were extracted. Details of case selection are presented in Supplementary Figure 1. Of note, immunosuppression was ascertained through a SIVEP-Gripe registry variable that lacks a standardized clinical definition and does not capture the type or severity of immunodeficiency, resulting in a heterogeneous exposure group. For context, the conditions defined by the Brazilian Ministry of Health early in the pandemic as indicative of high-grade immunosuppression are listed in the supplemental technical note.

In-hospital mortality was defined as death occurring during hospitalization (numerator) among all eligible cases (denominator), representing hospital case fatality rather than population-level mortality. Regarding COVID-19 vaccination, only vaccine doses with a recorded date of administration were considered valid (up to six vaccine doses analyzed).

Multivariable logistic regression was used to assess the association between COVID-19 vaccination and in-hospital COVID-19–related mortality (binary outcome). Covariates included in the model were selected based on clinical and epidemiological relevance, supported by prior literature and bivariate associations (p<0.10), provided that no multicollinearity was detected. Statistical analyses were performed using R software (version 4.5.0).

### Data availability statement:

All data used to prepare this analysis manuscript are publicly available at https://joaofelipe.github.io/COVID-Study/.

## RESULTS

A total of 474 immunocompromised older adults hospitalized for COVID-19 in Brazil during epidemiological weeks 1–52 of 2024 were included in the analysis (Table 1 and Supplementary Figure 1). Among them, 171 died, resulting in an in-hospital COVID-19-related case fatality of 36%. The median age was 75 years (range: 60–101); 53% were female, 66% self-identified as white, and 66% had at least one additional clinical risk factor for SARS.

**Table 1:** Bivariate analysis of baseline characteristics according to in-hospital outcome among immunocompromised older adults hospitalized with COVID-19 in Brazil, 2024 epidemiological year (SIVEP-Gripe database).

Characteristic		Survivor	Deceased	p-value*	Crude OR (95%CI)
Total number		303	171	NA	
Epidemiologic week of the onset of first COVID-19 symptoms, median (IQR)		12 (29)	14 (30.5)	0.46	1.00 (0.99–1.01)
Days from onset of first COVID-19 symptoms to hospitalization, median (IQR)		2 (3)	2 (5)	0.18	0.97 (0.93–1.01)
Age group, years, n (%)†				0.87	1.00 (0.98–1.02)
	60–69	103 (34)	61 (36)		(reference)
	70–79	101 (33)	53 (31)		0.89 (0.56–1.40)
	80+	99 (33)	57 (33)		0.97 (0.62–1.53)
Gender, n (%)					
	Female	156 (51)	96 (56)	0.38	(reference)
	Male	147 (49)	75 (44)		0.83 (0.57–1.21)
Race or Ethnicity, n (%)					
	White	202 (67)	109 (64)	0.34	(reference)
	Brown	54 (18)	43 (25)		1.48 (0.93–2.34)
	Black	7 (2)	3 (2)		0.79 (0.17–2.92)
	Yellow	3 (1)	1 (1)		0.62 (0.03–4.89)
	Unknown	37 (12)	15 (9)		0.75 (0.38–1.40)
Educational attainment, n (%)					
	High school or higher	62 (20)	19 (11)	0.01	(reference)
	Up to elementary school completed	50 (17)	44 (26)		2.87 (1.51–5.62)
	Up to middle school completed	20 (7)	18 (11)		2.94 (1.30–6.73)
	Unknown	171 (56)	90 (53)		1.72 (0.98–3.11)
	Other underlying risk conditions, n (%)‡	207 (68)	107 (63)	0.22	0.78 (0.52–1.15)
	Intensive care unit admission, n (%)	82 (27)	84 (49)	<0.001	2.60 (1.76–3.86)
	Invasive ventilatory support, n (%)	13 (4)	66 (39)	<0.001	14.02 (7.66–27.55)
COVID-19 vaccine doses, n (%)†				0.09	0.92 (0.82–1.04)
	Unvaccinated	32 (10.6)	24 (14)		(reference)
	1	6 (2)	1 (0.6)		0.22 (0.01–1.42)
	2	55 (18.2)	38 (22.2)		0.92 (0.47–1.81)
	3	63 (20.8)	32 (18.7)		0.68 (0.34–1.34)
	4	72 (23.8)	40 (23.4)		0.74 (0.38–1.43)
	5	70 (23.1)	35 (20.5)		0.67 (0.34–1.30)
	6	5 (1.7)	1 (0.6)		0.27 (0.01–1.80)
COVID-19 vaccination regimens, n (%)§					
	Inactivated vaccine only	60 (22.2)	27 (18.5)	0.30	0.59 (0.30–1.18)
	mRNA vaccine only	10 (3.7)	3 (2.1)		0.40 (0.08–1.45)
	Viral vector vaccine only	56 (20.7)	41 (28.1)		0.97 (0.50–1.87)
	Inactivated + mRNA vaccines	50 (18.5)	19 (13)		0.50 (0.24–1.05)
	Inactivated + viral vector vaccines	18 (6.7)	15 (10.3)		1.10 (0.46–2.60)
	mRNA + viral vector vaccines	44 (16.3)	22 (15.1)		0.66 (0.32–1.37)
	Inactivated + mRNA + viral vector vaccines	32 (11.9)	19 (13)		0.78 (0.36–1.69)
Interval since hospitalization and last COVID-19 vaccine dose, n (%)					
	Over one year	209 (77.1)	121 (82.3)	0.23	(reference)
	7–12 months	54 (19.9)	25 (17)		0.80 (0.47–1.34)
	Up to 6 months	8 (3)	1 (0.7)		0.22 (0.01–1.20)
	COVID-19 specific treatment, n (%)//	13 (4)	4 (2)	0.40	0.53 (0.15–1.54)

* Frequencies were compared using Pearson's χ^2^ or Fisher's exact test, as appropriate; continuous variables were assessed with the Mann-Whitney U test; trend across vaccine dose categories was evaluated with the Cochran-Armitage trend test

† In addition to categorical analyses, age and number of COVID-19 vaccine doses were modeled as continuous variables in univariable logistic regression

‡ Assessed conditions included diabetes mellitus, obesity, Down's syndrome, and chronic cardiovascular, pulmonary, hepatic, renal, hematologic, and neurologic diseases

§ The unvaccinated group was used as the reference category for crude odds ratio estimates. Information on the vaccine manufacturer was missing for one case record in each study group

// Included nirmatrelvir/ritonavir, molnupiravir, baricitinib, and remdesivir.

OR: odds ratio; 95%CI: 95% confidence interval; IQR: interquartile range; mRNA: messenger RNA.

Only 56 individuals (12%) were unvaccinated at the time of hospitalization. In bivariate (unadjusted) analyses, lower educational attainment, admission to an intensive care unit, and use of mechanical ventilation were significantly more frequent (p<0.05) among patients who died. In addition, a trend toward lower in-hospital COVID-19-related mortality was observed with increasing numbers of COVID-19 vaccine doses, although this did not reach statistical significance (p=0.09).

The multivariable logistic regression model, adjusted for age, sex, and covariates with p<0.10 in bivariate analyses, is presented in Table 2 and Supplementary Figure 2. After adjustment, invasive ventilatory support remained the strongest independent predictor of death (odds ratio [OR] 12.55; p<0.001), followed by lower educational attainment, defined as elementary or middle school education (OR 2.94 and 2.89, respectively; p<0.05 for both). COVID-19 vaccination showed a protective direction of effect, especially with four or more doses, although none of these associations reached statistical significance.

**Table 2:** Final multivariable logistic regression model evaluating the association between COVID-19 vaccination and in-hospital COVID-19-related mortality among immunocompromised older adults. Data from the Brazilian SIVEP-Gripe database, 2024 epidemiological year.

Characteristic		Odds ratio	95%CI	p-value
Age		1.01	0.99–1.03	0.42
Gender				
	Female (reference)	1.00		
	Male	1.07	0.70–1.65	0.75
Educational attainment				
	High school or higher (reference)	1.00		
	Elementary	2.94	1.39–6.44	0.01
	Middle	2.89	1.12–7.50	0.03
	Unknown level	1.76	0.92–3.51	0.10
Intensive care unit admission				
	No (reference)	1.00		
	Yes	1.44	0.87–2.37	0.15
Invasive ventilatory support				
	No (reference)	1.00		
	Yes	12.55	6.39–26.33	< 0.001
COVID-19 vaccine doses*				
	Unvaccinated (reference)	1.00		
	1–2	0.67	0.32–1.39	0.28
	3	0.67	0.31–1.42	0.30
	4	0.54	0.26–1.15	0.11
	5–6	0.56	0.26–1.17	0.12

* Categories for 1–2 and 5–6 COVID-19 vaccine doses were grouped due to the very small number of individuals who had received exactly 1 or 6 doses.

95%CI: 95% confidence interval.

## DISCUSSION

The study evaluated the association between COVID-19 vaccination and COVID-19–related mortality among immunocompromised older adults hospitalized for the disease, using data from the SIVEP-Gripe database. The in-hospital COVID-19-related mortality during epidemiological weeks 1–52 of 2024 was 36%, higher than that reported in the United States-based COVID-NET study conducted during earlier pandemic phases^6^, which used a comparable hospital-based surveillance design among immunocompromised patients.

Similar to the findings of the COVID-NET study^6^, COVID-19 vaccination was not significantly associated with a reduction in COVID-19–related mortality among immunocompromised individuals once hospitalized. Importantly, however, all adjusted ORs for vaccinated groups were below unity, suggesting a trend toward protection with increasing numbers of vaccine doses. No differences in mortality were observed across multiple COVID-19 vaccination regimens. Beyond vaccination status, lower educational attainment was independently associated with a higher likelihood of death, likely reflecting broader socioeconomic disadvantage.

The study, however, has limitations. First, reliance on administrative data restricted the availability of detailed clinical information. Second, waning immunity may have attenuated observable protective effects, since most vaccinees (79%) had received their last dose more than 12 months before hospitalization. Third, the analysis was limited to hospitalized cases, which may introduce selection and survival biases, as vaccinated individuals may have been less likely to require hospitalization. Accordingly, the findings pertain to in-hospital case fatality rather than overall COVID-19 mortality. In addition, the small number of unvaccinated individuals limited statistical power, and the high proportion of missing data on educational attainment may have reduced the precision of socioeconomic estimates.

Taken together, these findings suggest that the lack of a statistically significant association between COVID-19 vaccination and in-hospital COVID-19-related mortality among immunocompromised older adults may reflect, at least in part, the limited effectiveness of largely outdated vaccination regimens in a population characterized by advanced age, immunocompromise, and severe clinical presentation, rather than the absence of vaccine benefit. In this context, the results underscore the need for additional protective strategies for this vulnerable population, including timely booster vaccination, early diagnosis, and optimized preventive and therapeutic interventions.
