# Supplementary material for: COVID-19 vaccine effectiveness against in-hospital mortality in immunocompromised older adults: a Brazilian hospital surveillance study, 2024
Source: Rev Bras Epidemiol. 2026 Aug 17;29:e260041. doi: 10.1590/1980-549720260041 (PMC13480620; doi:10.1590/1980-549720260041)
Supplement: Supplementary file 1 [file 1980-5497-rbepid-29-e260041-Suppl01.pdf]

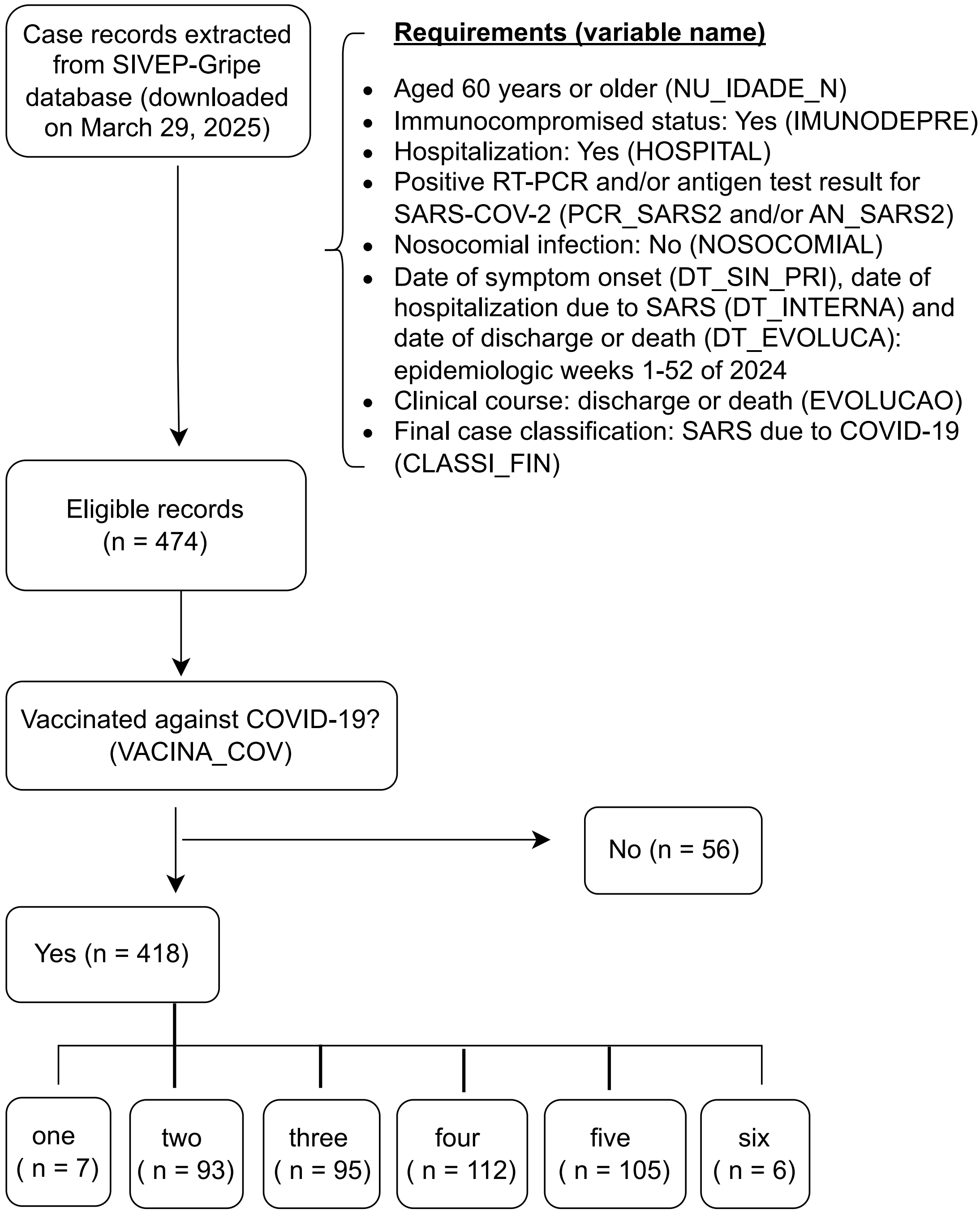

Supplementary Figure 1. Flowchart of case selection from the SIVEP-Gripe database.

Abbreviations: RT-PCR, reverse transcription polymerase chain reaction; SARS, severe acute respiratory syndrome; SARS-CoV-2, severe acute respiratory syndrome coronavirus 2; SIVEP-Gripe, Sistema de Informação da Vigilância Epidemiológica da Gripe (Brazilian Influenza Surveillance Information System).
